# Metabolomic profiling of host–pathogen interactions: differential effects of Gram-positive and Gram-negative bacterial secretomes on THP-1 macrophage metabolism

**DOI:** 10.1039/d4ra07202b

**Published:** 2025-10-22

**Authors:** Alaa Abuawad, Manuel Romero, Sandra Martinez Jarquin, Amir M. Ghaemmaghami, Dong-Hyun Kim

**Affiliations:** a Department of Pharmaceutical Sciences and Pharmaceutics, Faculty of Pharmacy, Applied Science Private University Amman Jordan; b National Biofilms Innovation Centre, Biodiscovery Institute and School of Life Sciences, University of Nottingham Nottingham UK; c Centre for Analytical Bioscience, Advanced Materials and Healthcare Technologies Division, School of Pharmacy, University of Nottingham Nottingham NG7 2RD UK dong-hyun.Kim@nottingham.ac.uk +44 (0) 115 74 84697; d Immunology & Immuno-bioengineering Group, School of Life Sciences, Faculty of Medicine and Health Sciences, University of Nottingham Nottingham NG7 2RD UK; e College of Pharmacy, Kyungpook National University Daegu 41566 Republic of Korea

## Abstract

Infectious diseases present substantial health and economic challenges worldwide. The increasing prevalence of multidrug-resistant bacteria in both community and hospital settings has emerged as a global health issue that necessitates innovative strategies for prompt diagnosis and treatment. Metabolomics, which provides comprehensive insights into the biochemical alterations of cellular phenotypes, has emerged as a valuable approach for studying host–pathogen interactions and identifying novel therapeutic targets. In this study, untargeted liquid chromatography-mass spectrometry (LC-MS)-based metabolite profiling was employed to investigate the differential effects of the secretome from Gram-positive *S. aureus* SH1000 and Gram-negative *P. aeruginosa* PAO1 on THP-1 macrophages. The results revealed that both bacterial secretomes modulate several key metabolic pathways, including alanine, aspartate and glutamate metabolism; sphingolipid metabolism; glycine and serine metabolism; glycolipid metabolism; and tryptophan metabolism. Distinct metabolic trends were observed between the two secretomes: *S. aureus* induced an accumulation of asparagine and l-formylkynurenine, alongside depletion of glycine-related intermediates (*e.g.* sarcosine, guanidinoacetate), whereas *P. aeruginosa* altered creatine levels and reduced asparagine and l-kynurenine. Notably, shared effects were also identified, with both secretomes demonstrating similar significant effects (FDR < 0.05 and VIP > 1) on arginine and proline metabolism in THP-1 macrophages. These findings highlight both shared and unique pathogen-specific metabolic responses, offering preliminary insights into host metabolic reprogramming triggered by exemplar Gram-positive and Gram-negative bacteria. These results provide a foundation for future studies to explore bacterial pathogenesis and to identify therapeutic strategies against resistant infections.

## Abstract 2

Treating THP-1 macrophages with *S. aureus* or *P. aeruginosa* secretome showed pronounced and distinctive metabolic disturbance.

## Graphical abstract

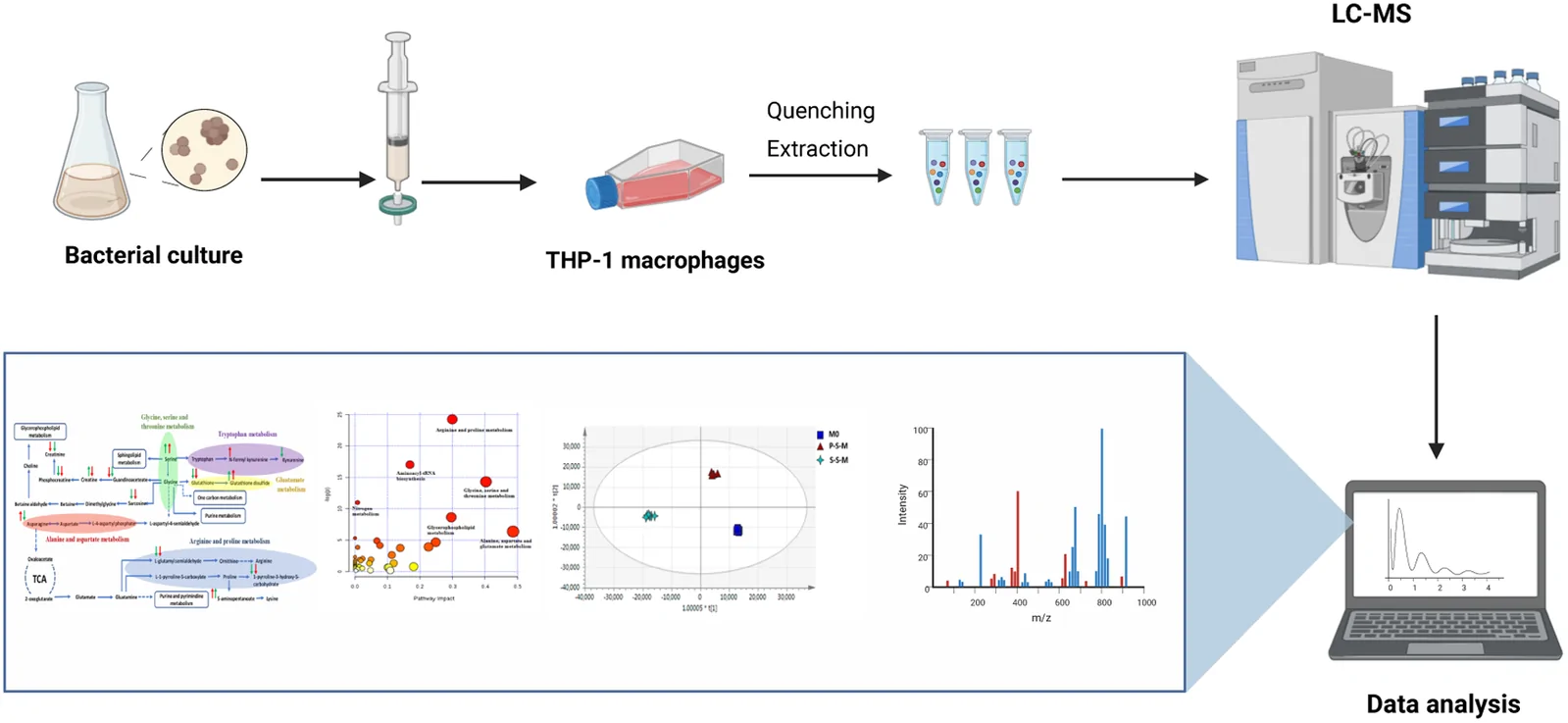

## Introduction

Infectious diseases represent major health and economic challenges worldwide and have constantly featured amongst the top 10 causes of death globally.^1,2^ The emergence of multiple drug-resistant bacteria in the community and hospital has become a worldwide concern, as resistant bacterial infections are associated with longer hospitalisation and an increase in the mortality rate.^3^ Therefore, there is an urgent need for innovative and more efficient diagnostic and treatment approaches.^4^

*Staphylococcus aureus* (*S. aureus*), an archetypal Gram-positive bacterium, represents a major human pathogen. It is an opportunistic bacterium normally colonising the skin and the respiratory tract causing a wide range of infectious diseases.^5^ On the other hand, *Pseudomonas aeruginosa* (*P. aeruginosa*) is an opportunistic Gram-negative bacterium that can cause a serious infection in individuals with compromised immune defence systems. They are leading causes of many serious diseases such as bacteraemia, skin and soft tissue infections, urinary tract infections and respiratory tract infections.^6^ Given the severity of the infections caused by these two types of bacteria and the antibiotic resistance they display, there has been a substantial interest in understanding their pathobiology. A few studies have shown that the pathogenicity of bacteria is multifactorial and caused by various virulence factors such as toxins, enzymes, and adhesins, as well as the production of secondary metabolites.^7,8^ A wide range of virulence factors enable the bacteria to avoid the immune system. Nevertheless, the immune system can recognise different features of pathogens by specific surface receptors called pathogen recognition receptors (PRRs). For instance, Toll-like receptors (TLRs) play a crucial role in triggering immune responses against bacteria.^9^ Historically, immunology has predominantly focused on immune signalling pathways due to their pivotal role in activating and regulating immune cells in response to pathogens. Early research prioritised understanding the complex mechanisms involving cytokines and surface receptors, leading to a substantial body of knowledge on these interactions. In contrast, the field of immunometabolism, which explores the relationship between metabolic processes and the function and fate of immune cells, has only recently gained significant attention. Emerging studies have revealed the profound metabolic changes immune cells undergo to meet the energy and biosynthetic demands of activation and differentiation.^10^ Despite its importance, immunometabolism remains less explored compared to signalling pathways. This disparity stems from the complexity of the numerous metabolic pathways involved, coupled with the fact that each immune cell type exhibits distinct metabolic profiles that vary dynamically depending on stimulation and environmental cues.^10,11^ Furthermore, immunometabolism is an inherently interdisciplinary field, requiring the integration of diverse disciplines, which presents unique challenges in research design and methodology.^12^

Macrophages are effector immune cells that play a critical role in innate and adaptive immunity^13^ and are characterised by the expression of a wide range of PRRs, most popularly, TLRs.^9,13^ Lipopolysaccharide (LPS), a component of the outer membrane of Gram-negative bacteria, is recognised by TLR4, whereas lipoteichoic acid (LTA) and peptidoglycan (PG) from the Gram-positive bacterial cell wall are recognised by TLR2.^14^ Given that Gram-negative and Gram-positive bacteria engage with different sets of TLRs that are linked to different signalling pathways, it is not surprising that different bacterial infections have distinct immunological profiles. However, less is known about the metabolic responses of immune cells to different types of a bacterial infection.

The emergence of antibiotic-resistant bacteria in both hospital and community settings urges the development of innovative approaches for diagnosis and treatment. Currently, culturing microorganisms from blood or other body fluids is the most used for bacterial infection diagnosis. Molecular tests have been increasingly used in clinical laboratories, but not as a part of routine practice due to the high cost. However, routine tests, while cheaper, are time-consuming with low accuracy and sensitivity which could provide erroneous information, especially in patients with immunodeficiency.^15^ These drawbacks of routine tests have led to an increased effort to identify disease-specific biomarkers that can be used in the prediction of the nature and severity of bacterial infections. Such biomarkers can potentially lead to early diagnosis and selection of the optimal treatment at an early stage, hence improving health outcomes.^16^ For instance, Wunderink *et al.* demonstrated that procalcitonin is mainly produced in response to bacterial infections and is considered to be a diagnostic biomarker to differentiate between viral and bacterial infections.^17^ However, a single metabolite biomarker can be an intermediate in more than one metabolic pathway. Moreover, the most challenging mission is to differentiate between infectious diseases caused by different strains of bacteria. Therefore, novel approaches are needed to improve the microbiological readout. In this context, in the last few years, the research has moved from focusing on a single biomarker to a biosignature encompassing a set of reliable markers.^18^

Liquid chromatography (LC)-mass spectrometry (MS)-based metabolomics has been used widely for disease diagnostic biomarker discovery due to its sensitivity and powerful quantification ability of low molecular metabolites in biological samples.^19^ The technique has been successfully employed in bacterial infection studies by discovering the biomarkers and the changes in the metabolic pathways upon infection.^20,21^ For example, Müller *et al.* applied non-targeted metabolomics to HEP-2 cells in response to *Chlamydia pneumoniae* infection, demonstrating significant alteration in various metabolisms upon infection which could be potential biomarkers for diagnosis and drug target.^20^ In another metabolomics study, Antunes *et al.* investigated the impact of *Salmonella* on the murine immunological response, revealing that many metabolites significantly changed in response to *Salmonella* infection.^22^ Furthermore, Fischer *et al.* successfully employed LC-MS-based metabolomics to identify biomarkers for decompensated cirrhosis, particularly in patients with overlapping bacterial infections. Their study revealed that the primary metabolic pathways affected in decompensated cirrhosis were those related to lipid metabolism, with *N*-oleoyl ethanolamine emerging as the most promising biomarker for diagnosing bacterial infections.^23^ In another metabolomics study, Liu *et al.* profiled serum samples from syphilis patients, and identified trimethylamine *N*-oxide as a potential biomarker for syphilis diagnosis.^24^

Due to the crucial pro-inflammatory role of macrophages in fighting bacterial infections,^13^ investigation of macrophages response to bacterial extracellular secreted molecules (secretome) using a metabolomics approach may have a dual benefit in the study of bacterial infections: (i) in a diagnostic role by revealing potential biomarkers and (ii) by increasing the chance for novel therapeutics by targeting relevant pathways.

Unlike conventional research that primarily investigates whole bacteria or intracellular factors, our study focuses on the bacterial secretome, encompassing the extracellular factors that directly impact host cell profiles. This approach allows us to examine the unique metabolic and immune responses elicited by extracellular components.

In contrast to previous studies that often generalise findings across bacterial types, our research identifies distinct metabolic pathways altered by Gram-positive *S. aureus* and Gram-negative *P. aeruginosa*. Specifically, we highlight how the unique receptor pathways of TLR2 and TLR4, respectively, drive differential immune responses and metabolic perturbations.^25,26^ These findings provide a more accurate representation of macrophage polarisation during infection, which differs significantly from polarisation induced by lipopolysaccharide (LPS) alone into a pro-inflammatory phenotype.^27,28^

Additionally, our study employs untargeted metabolic profiling to explore the immunometabolism of these infections, addressing challenges inherent in this approach. While most previous studies have used alternative models rather than immune cells^20,22–24^ or have relied on targeted metabolomics that assess a limited number of metabolites,^27^ our work provides a comprehensive analysis of metabolic alterations during bacterial infections.

In this study, we performed LC-MS-based metabolite profiling to investigate the impact of the secretome from the spent culture media of the Gram-positive *S. aureus* SH1000 and Gram-negative *P. aeruginosa* PAO1 on THP-1 macrophages. This approach has the potential to provide novel diagnostic insights and may facilitate the development of new therapeutic strategies for bacterial infections.

## Materials and methods

### Materials

THP-1 cell line was purchased from ATCC, USA. RPMI-1640 medium foetal bovine serum (FBS), phosphate buffered saline (PBS), 2 mM l-glutamine, 100 U ml^−1^ penicillin, 100 μg ml^−1^ streptomycin and tryptic soy broth (TSB) were purchased from Sigma-Aldrich, UK. 0.2 μm sterilised filters were purchased from Sartorius, UK. Methanol and acetonitrile were purchased from Fisher Scientific, UK. All solvents were LC-MS grade.

## Methods

### Cell culture and differentiation

Human THP-1 cells were cultured and differentiated as previously described.^29^ Briefly, THP-1 cells were grown in T75 tissue culture flasks using RPMI 1640 supplemented with 10% heat-inactivated fetal bovine serum (FBS), 1% l-glutamine, and 1% penicillin-streptomycin. Cells were then incubated at 37 °C and 5% CO_2_. THP-1 cells were differentiated into naïve macrophage states (M0) by treatment with phorbol-12-myristate-13-acetate (PMA) at a final concentration of 50 ng ml^−1^. Macrophage hallmarks and morphology were examined as described previously.^30^ Six million cells were seeded per flask (T25 tissue culture flask), and then the cells were treated with six ml of PMA-containing media with a final concentration of 50 ng ml^−1^ of PMA and incubated for 24 h.

### Bacterial culture and cell treatment with bacterial secretome

*P. aeruginosa* PAO1, *P.* and *S. aureus* SH1000 in tryptic soy broth (TSB) were grown in 50 ml of culture media in 250 ml conical flasks with shaking (200 rpm) at 37 °C. After 18 h of incubation, spent culture media were centrifuged, and supernatants were recovered and filter sterilised. 10% (600 μl) of spent culture media for each bacteria (*S. aureus* SH1000 and WT *P. aeruginosa* PAO1) were used to treat macrophages with incubation for 24 h (henceforth referred to as S-S-M and P-S-M, respectively). Untreated M0 was set as a control. Six biological replicates of each condition were prepared.

### Sample preparation and metabolite extraction

After incubation for 24 h, the media were removed. The cells were then washed once with pre-warmed PBS (37 °C) and 500 μl pre-cooled methanol at −48 °C using dry ice was used for quenching and metabolite extraction. The cells were harvested using a plastic scraper whilst being kept on ice and the extracts were transferred into pre-cooled fresh 2 ml tubes (4 °C). The cell extracts were vortexed for 1 h at 4 °C and centrifuged at 16 100×*g* for 10 min at 4 °C. After the centrifugation, the supernatants were dried under vacuum and reconstituted with 70 μl methanol, and then stored at −80 °C prior to LC-MS analysis. To assess the instrument performance, a quality control (QC) sample was prepared by mixing an equal volume of all the samples and PCA analysis was performed to ensure the system suitability (SI Fig. 1).

### Analytical methodologies

LC-MS-based metabolite profiling was performed on an Accela system coupled to an Exactive MS (Thermo Fisher Scientific, Hemel Hempstead, UK) operating with electrospray ionisation (ESI) running in the negative (ESI−) and positive (ESI+) modes as previously described in.^31^ Briefly, the spray voltage was 4500 V (ESI+) and 3500 V (ESI−), the capillary voltage was 40 V (ESI+) and 30 V (ESI−), and tube lens voltage was 70 V for both modes and skimmer voltage was 20 V (ESI+) and 18 V (ESI−). The temperature for capillary and probe was maintained at 275 °C and 150 °C, respectively. Chromatographic separation was carried out using ZIC-pHILIC (4.6 × 150 mm and 5 μm particle size, Merck Sequant). The mobile phase was composed of 20 mM ammonium carbonate in water (solvent A) and 100% acetonitrile (solvent B). Metabolites were separated according to a linear gradient as follows: 0–15 min (20% A), 15–17 min (95% A), and 17–24 min (20% A) at 300 μl min^−1^ flow rate. The injection volume was 10 μl and the column was kept at 45 °C.

### Data processing and metabolite identification

To process raw data obtained from LC-MS (with representative total ion chromatograms shown in SI Fig. 2), XCMS and mzMatch were used for untargeted peak-picking and peak matching, respectively.^32,33^ IDEOM was performed for putative metabolite identification and noise filtering with default parameters.^34^ Briefly, RT for identification of authentic standards was 5%, RT for identification for calculated RT was 50%, ppm for mass identification was 3 ppm. Metabolites were identified with four levels of confidence; level 1 (L1) identification was based on matching the accurate masses, MS/MS fragmentation and retention times of the detected metabolite peaks with those of 250 authentic standards which were co-analysed with the samples under identical experimental conditions, level 2 (L2) identification was based on matching the accurate masses and retention times (two orthogonal data) of the detected metabolite peaks with those of the authentic standards, level 3 (L3) identification was carried out when the predicted retention times were employed due to the lack of standards and level 4 (L4) identification was based on unambiguously assigned molecular formulas but insufficient evidence exists to propose possible structures. The identification criteria were according to the metabolomics standards initiative.^35–37^

Pre-processed data were analysed by performing multivariate and univariate analysis. OPLS-DA was carried out by SIMCA-P v13.0.2 (Umetrics, Umea, Sweden) as a supervised multivariate model. This multivariate analysis was used as the first step for visualising data with sample classes and evaluating the metabolome differences between M0 and *P. aeruginosa* or *S. aureus*. Cross-validation is a key method used to evaluate the performance and reliability of a model. In SIMCA, this process is summarised through various quality metrics, with *R*^2^ and *Q*^2^ being the most widely used in metabolomics. Both metrics range from 0 to 1, with *R*^2^ = 1 signifying a perfect fit of the model to the data and *Q*^2^ = 1 indicating flawless predictive ability. For a model to be considered robust, it is generally recommended that both *R*^2^ and *Q*^2^ exceed 0.5.^38^ Additionally, the OPLS-DA models were validated using a permutation test. The key mass ions representing potential biomarkers were determined based on their variable importance of projection (VIP) values obtained from two-way orthogonal comparisons. Mass ions with VIP values greater than one were considered as discriminant biomarkers. Univariate analysis was also performed in parallel with multivariate analysis to identify significant mass ions. *T*-test with FDR correction was performed using Metaboanalyst^39^ to determine the significantly changed mass ions between M0 and S-S-M or P-S-M. MetaboAnalayst and KEGG database were used to analyse and visualise the affected pathway.

## Results and discussion

### Characterisation of macrophages

To visualise cell morphology, differentiated THP-1 cells were stained with DAPI for nuclear visualisation and fluorescently labelled phalloidin to detect F-actin filaments. Immunofluorescence staining was also performed to assess the expression of surface markers calprotectin and mannose receptor (MR). Consistent with previously reported morphological characteristics, the cells exhibited a rounded macrophage-like shape (SI Fig. 3A) and expressed both calprotectin and MR (Fig. 3B), confirming successful differentiation into naïve (M0) macrophage states.^30^

### Multivariate analysis

**Fig. 1:**
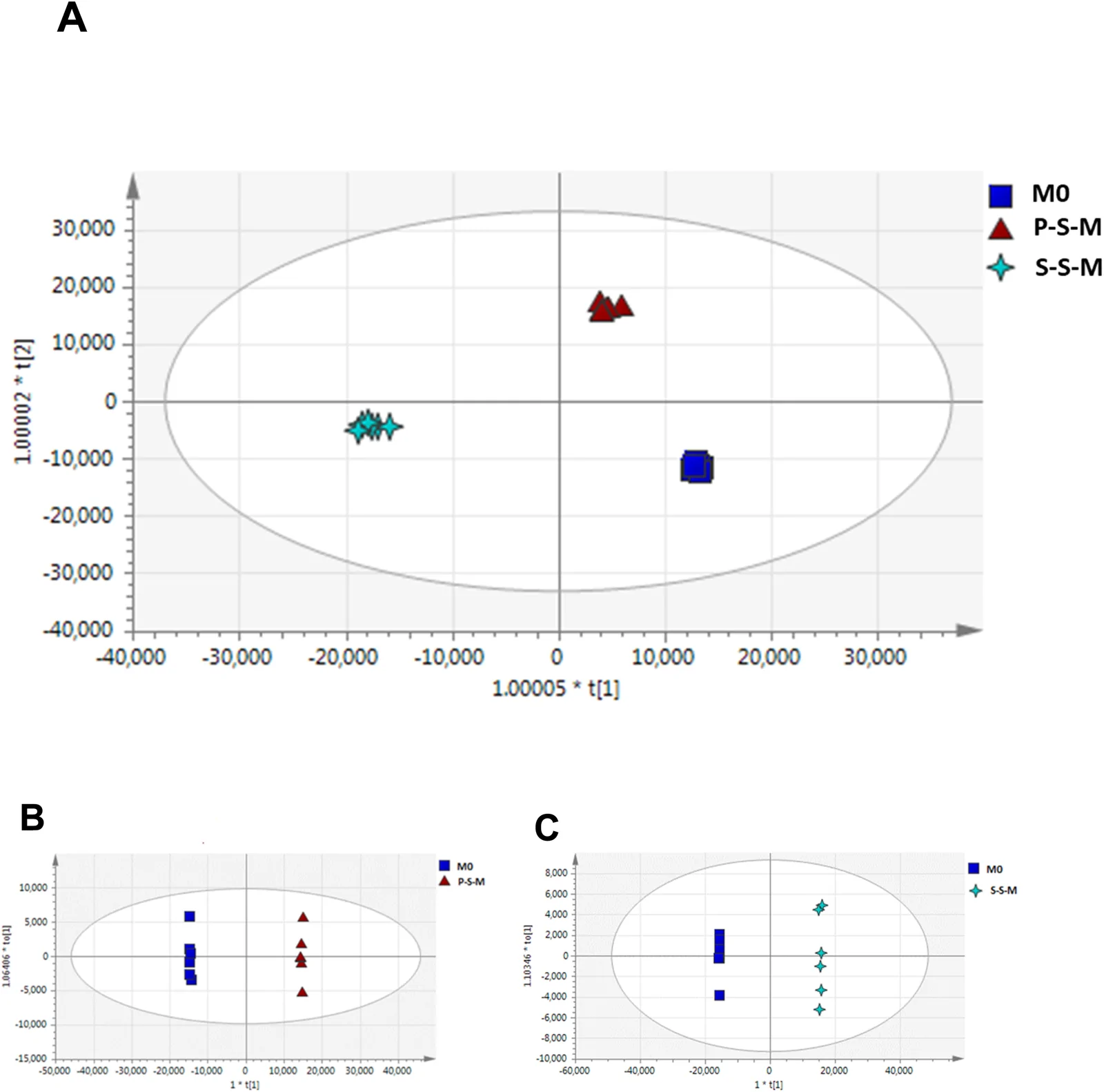
OPLS-DA scores plots of macrophage extracts after the treatment with the sectretome of *S. aureus* (S-S-M) and *P. aeruginosa* (P-S-M), and their corresponding untreated control (M0). (A) OPLS-DA scores plot of S-S-M, P-S-M and M0 (*R*^2^ = 0.863 and *Q*^2^ = 0.996). (B) Two-way orthogonal comparison between P-S-M and untreated M0 (*R*^2^ = 0.838 and *Q*^2^ = 0.999). (C) Two-way orthogonal comparison between S-S-M and untreated M0 (*R*^2^ = 0.842 and *Q*^2^ = 0.998). M0 (dark blue squares), P-S-M (red triangles), and *S. aureus* (light blue stars) *n* = 6.

In order to investigate and visualise the inherent metabolic differences between the macrophages challenged with the bacterial secretomes (S-S-M or P-S-M), OPLS-DA was performed for comparison with the untreated macrophages as a control (M0). In Fig. 1A, the OPLS-DA scores plot shows a tight clustering of six replicates within each group. Furthermore, a clear separation is shown between M0 and S-S-M or P-S-M with *R*^2^ and *Q*^2^ values of 0.863 and 0.996, respectively, demonstrating an acceptable and valid model.^38^ This result shows two important points: firstly, there is a change in the metabolic profiles of macrophages upon the exposure to the bacterial secretome and secondly, there is a clear separation between S-S-M and P-S-M. These findings reflect host metabolic responses to the specific secretomes tested in this study, rather than generalised Gram-positive *versus* Gram-negative signatures. To investigate which metabolites contributed to the separation between groups, two-way comparisons of OPLS-DA were performed as shown in Fig. 1B and C. The features were ranked according to their VIP values and those with VIP > 1 were considered as potential biomarkers. Multivariate analysis was accompanied by *t*-test with FDR correction. Most significantly changed features with VIP > 1 and FDR < 0.05 were selected as potential characteristic metabolites.

As can be seen in Fig. 1B, the OPLS-DA scores plot shows a clear separation between M0 and P-S-M with *R*^2^ and *Q*^2^ values of 0.848 and 0.999, respectively. A permutation test confirmed the validity of the constructed model (SI Fig. 4A). Of 501 identified metabolites (SI Table 1), 75 metabolites were significantly changed in response to the exposure of the secretome from *P. aeruginosa* (SI Table 2) based on the criteria (*i.e.* VIP > 1 and FDR < 0.05). Similarly, in Fig. 1C, the OPLS-DA scores plot shows a clear separation between M0 and S-S-M with *R*^2^ and *Q*^2^ values of 0.846 and 0.998, respectively, demonstrating an acceptable and valid model.^38^ Further validation using a permutation test showed the validity of the constructed models (SI Fig. 4B). Based on the combination of multivariate and univariate analyses, 83 metabolites (VIP > 1 and FDR < 0.05) were identified as key metabolites in response to the exposure of the secretome from *S. aureus* (SI Table 3). Building on the uni- and multivariate analyses that identified key metabolites for P-S-M and S-S-M, the data were imported into MetaboAnalyst for pathway analysis to determine the metabolic pathways involved in each condition.

### Pathway analysis

**Fig. 2:**
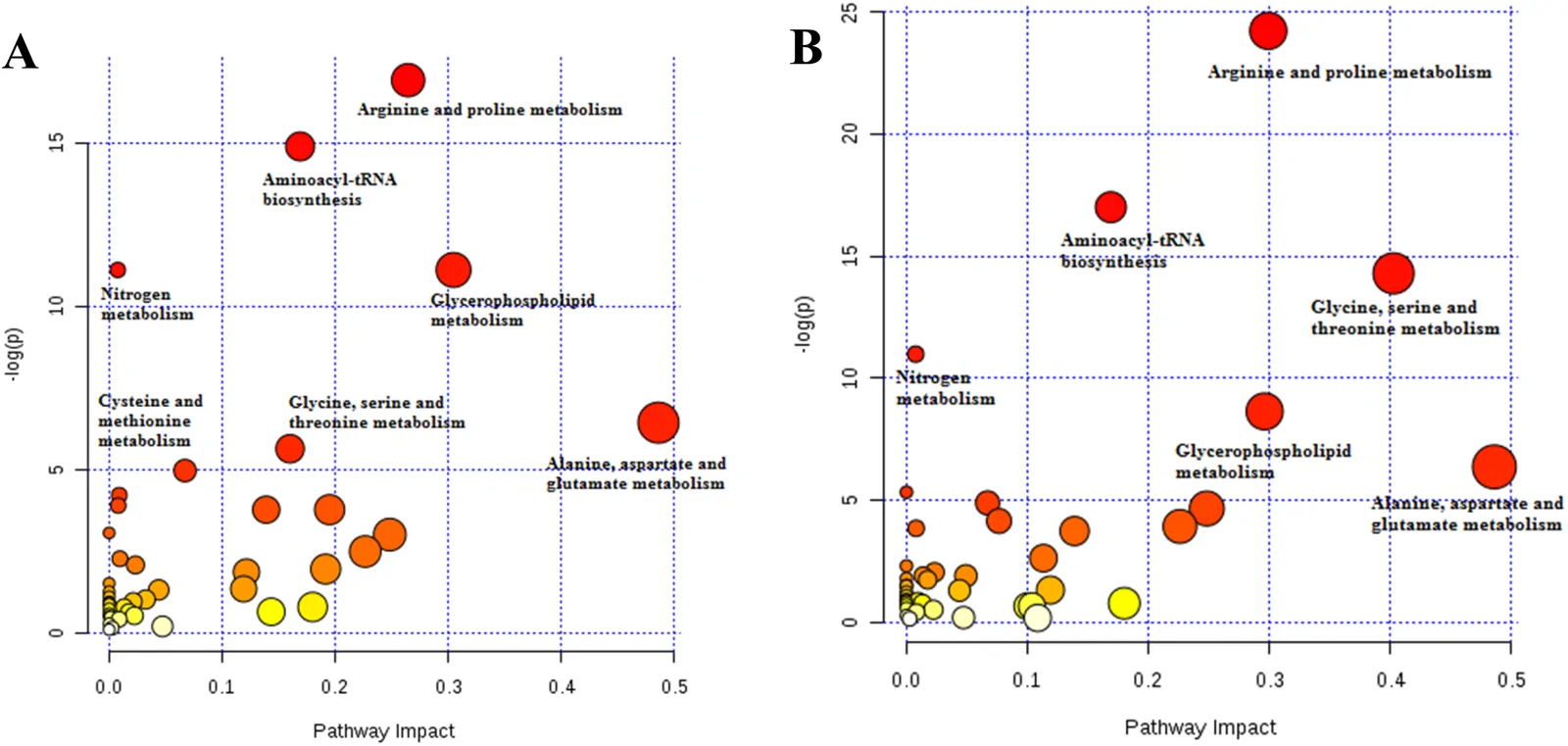
A summary of pathway analysis by MetaboAnalyst. The top pathways were ranked by the gamma-adjusted *p*-values for permutation per pathway (*Y*-axis) and the total number of hits per pathway (*X*-axis). The colour graduated from white to yellow, orange and red as the values of both x and y increase, red circles the most significantly changed metabolic pathway, and the pale yellowish are the least significantly changed metabolites. (A) Metabolic pathways significantly changed in *P. aeruginosa* sectretome treated macrophages. (B) Metabolic pathways significantly changed in *S. aureus* sectretome treated macrophages.

To further identify the metabolic pathways that were significantly altered upon the exposure of the bacterial secretome, a comprehensive pathway analysis was performed using MetaboAnalyst, as illustrated in Fig. 2A and B. These data show that the significant number of altered metabolites caused by the exposure to the bacterial secretome are relevant to amino acid metabolism as can be clearly seen in Fig. 2. Amino acid metabolism is very important for the host cells to enhance the immune defence against pathogens. l-tryptophan, l-arginine and l-asparagine are the main amino acids which the pathogen competes with the host cells for.^40^ Disturbance of amino acid pathways has been noticed as a cell adaptation in bacterial infections. Therefore, targeting these pathways in the host has recently been highlighted as a novel approach to manipulate bacterial infections.^41^

**Fig. 3:**
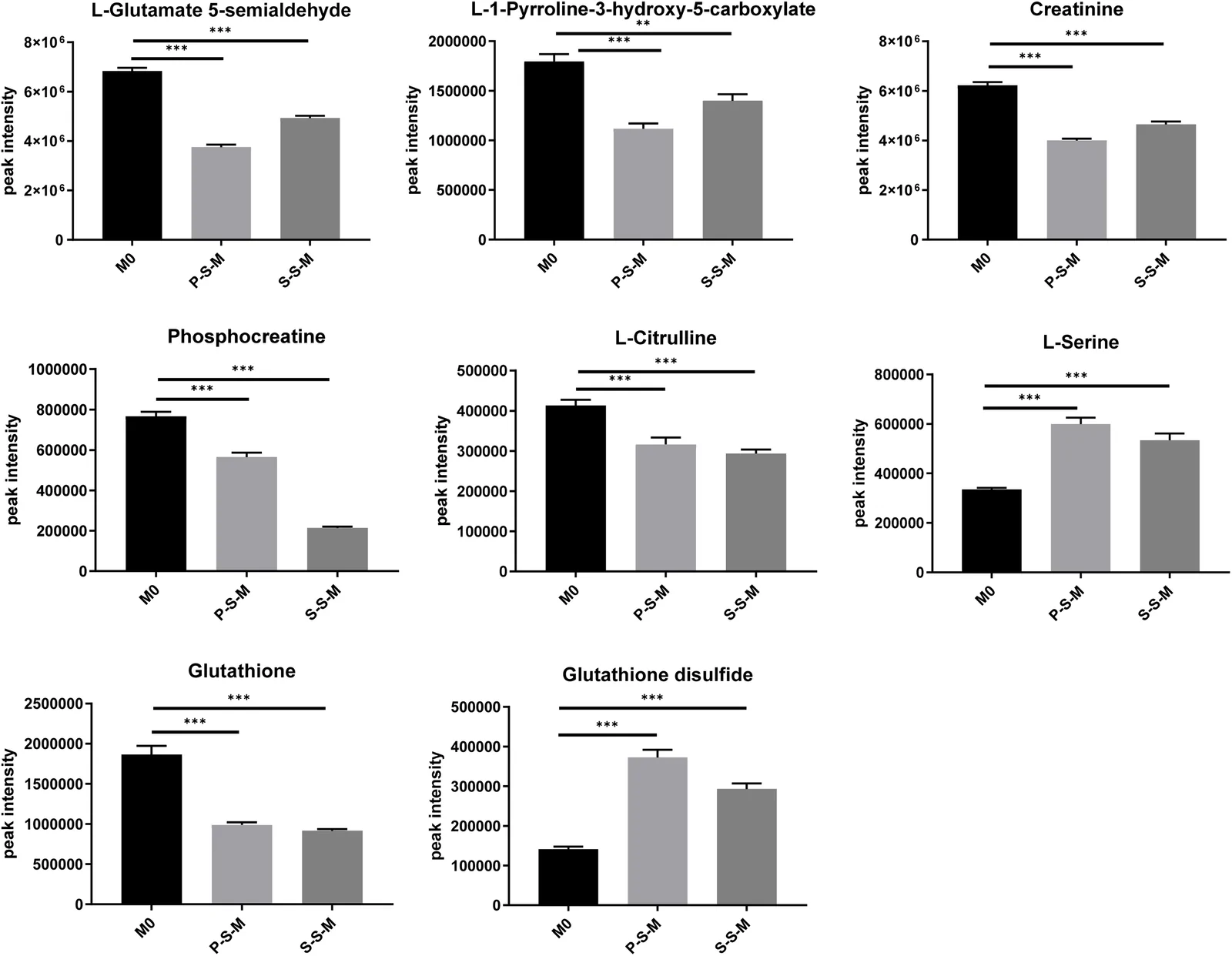
Significantly changed metabolites involved in amino acid metabolism in both P-S-M and S-S-M with the same trend (decrease or increase). l-1-Pyrroline-3-hydroxy-5-carboxylate, creatinine, l-glutamate 5-semialdehyde, l-citrulline and phosphocreatine represent arginine and proline metabolism. l-Serine from glycine, serine and threonine metabolism. *n* = 6, data are presented as mean ± SEM. Statistical significance was assessed using FDR correction. ns: not significant (*p* < 0.05), **p < 0.01, ***p < 0.001).

The arginine and proline metabolism pathway in Fig. 2A and B showed the most significant alteration in response to the exposure of the secretomes from *S. aureus* and *P. aeruginosa* infection. This could be due to the significant decrease in the level of l-1-pyrroline-3-hydroxy-5-carboxylate, creatinine, l-glutamate 5-semialdehyde, l-citrulline and phosphocreatine, which are intermediates in the arginine and proline metabolism, in both *S. aureus* and *P. aeruginosa* secretomes treated macrophages (Fig. 3). l-Arginine is considered a non-essential amino acid at the whole organism level, but it is important to be supplemented in certain diseases such as gastritis, ulcers, hypertension, and cardiovascular diseases. It is produced from *de novo* arginine biosynthesis or cellular protein breakdown.^42^ Metabolic flexibility and interconvertibility are well recognised for l-arginine since it can be interconverted with a range of other amino acids such as l-glutamate and l-proline. In addition, it is involved in the synthesis of metabolites such as creatine, phosphocreatine, polyamines, nitric oxide and urea cycle metabolites.^43^ Normally, l-arginine is a substrate of two main enzymes, arginase and inducible type 2 nitric oxide synthase. It can be metabolised by arginase to produce ornithine and urea or oxidised by iNOS to produce l-citrulline and nitric oxide (NO). Therefore, the competition of arginase with the iNOS is well studied for its activity in decreasing the production of NO. In addition to this competition, arginase was reported to inhibit the expression of iNOS, again limiting the production of NO.^43^ NO is a pivotal element of the immune response and is one of the most important antimicrobial agents of the host's first line of defence.^44^ Pathogenic bacteria adapt several mechanisms targeting arginine to protect itself against the immune system of a host cell. For instance, arginase diverts arginine away from iNOS to subvert an antimicrobial effect of macrophages.^40^ Moreover, pathogens upregulate the activity of arginase to decrease the availability of arginine as a substrate for iNOS. The arginase produced by bacteria can also similarly compete with iNOS to mammalian arginase to prevent NO production as a strategy of the bacteria to survive.^43^ The bacterial arginase was reported to be recruited to deplete l-arginine in macrophages.^45^ The depletion of l-arginine will consequently decrease the production of l-ornithine which is hydrolysed by ornithine aminotransferase followed by 1-pyrroline-5-carboxylate dehydrogenase to produce l-glutamate. Also, l-ornithine produces l-citrulline by ornithine carbamoyl transferase. A similar effect on the arginine and proline metabolism pathway was expected, as both *S. aureus* and *P. aeruginosa* have been reported to predominantly induce the expression of arginase in host cells and tissue over iNOS. This activation of arginase enhances the metabolism of l-arginine through the arginase pathway, thereby reducing the availability of l-arginine as a substrate for iNOS. Consequently, this limits the production of NO.^46–48^ Targeting the precursors such as l-arginine and l-ornithine by bacteria can explain the decrease in the level of intermediates such as l-citrulline, l-glutamate and other relevant metabolites (l-1-pyrroline-3-hydroxy-5-carboxylate, creatinine, l-glutamate 5-semialdehyde and phosphocreatine). This represented a potential pathway to target therapeutically by inhibiting arginase and increasing NO production to combat the infections with *S. aureous* and *P. aeruginosa*. This was successfully applied by Mehl. *et al.* where they demonstrated an increase in the production of NO due to inhibiting arginase during *P. aeruginosa* pneumonia infection in mice,^47^ Pang *et al.* also showed that enhanced expression of arginase has been inhibited by the metabolite biomarkers resulting in killing of *S. aureus*, in addition to enhance the metabolite-induced phagocytic activity against *S. aureus*.^46^ Glutathione is the most abundant low molecular weight thiol, and it plays a crucial role in confronting the oxidative stress of the cells resulting from bacterial infection-induced ROS production. The ratio of glutathione and glutathione disulphide (the oxidized form) represents the main redox balance in human cells.^49^ Our results showed that glutathione was significantly decreased whereas glutathione disulphide significantly increased in response to the exposure of the secretomes from *S. aureus* and *P. aeruginosa* (Fig. 3). This perturbation in the redox state indicates the oxidative stress condition of infected macrophages.^50^ Also, the one-carbon pathway has emerged as a key metabolic pathway in cell proliferation and immune functions. However, maintaining the redox balance and sustaining immune cell proliferation are crucial to confront oxidative stress or hypoxia that can be induced in the case of bacterial infections. It contributes to the production of NADPH and glutathione as it provides a source of the methyl group required for the synthesis of glutathione and purines, and DNA methylation.^51^ The production of glutathione from glycine occurs in all cell types.^52^ The significant decrease in glycine (SI Tables 2 and 3) could explain the significant reduction of glutathione, indicating the redox imbalance and oxidative stress condition. A reduction in the availability of glycine was reported as a result of inflammation stimuli.^53^ Also, Fang *et al.* suggested that glycine may become a limiting factor for the synthesis of glutathione.^49^

Glycine, serine, and threonine metabolism were amongst the most significantly altered amino acids upon the treatment with the secretomes from *S. aureus* and *P. aeruginosa* (Fig. 2A and B). Glycine and serine are non-essential amino acids that are involved in the synthesis of nucleic acids, proteins, and lipids as precursors for these processes. Serine is converted to glycine using the enzyme, serine hydroxymethyltransferase.^51^ This reaction plays a crucial role in the one-carbon pathway by supplying a methyl group required for the synthesis of proteins, glutathione and nucleotides. In a recent study, Ma *et al.* demonstrated that serine availability can serve as a checkpoint metabolite, indicating efficient immune response of primary T cells^54^ which emphasises the pivotal role of this pathway in the immune response against challenging pathogens such as bacteria. Our data showed significant elevation of serine in S-S-M and P-S-M (Fig. 3). Glycine can potentially enter the folate cycle by donating one carbon through the glycine cleavage process.^51^

**Fig. 4:**
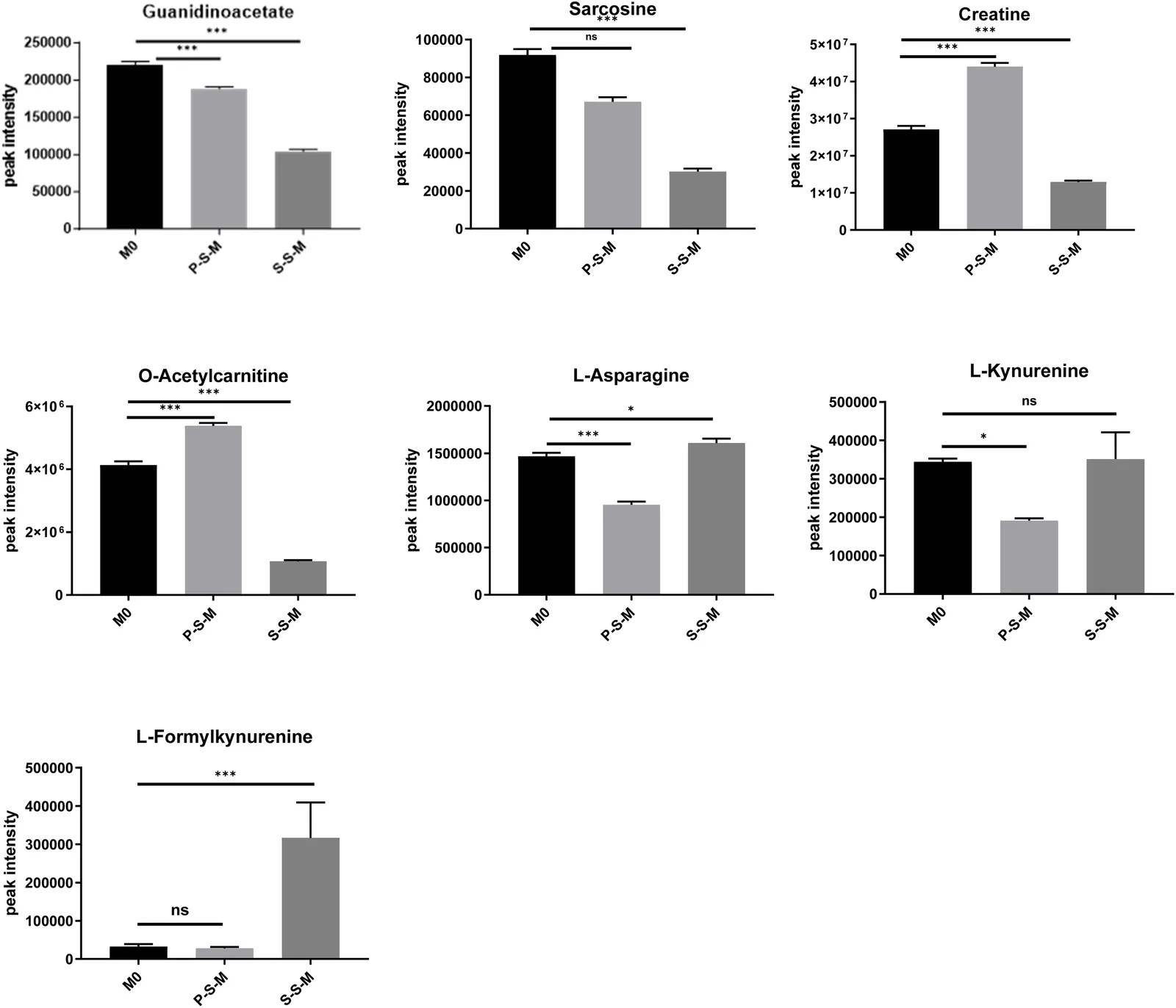
Amino acids relevant differentially and significantly changed metabolites in P-S-M and S-S-M. Sarcosine, guanidinoacetate and creatine represent glycine, serine and threonine metabolism. *O*-Acetylcarnitine, and l-asparagine represent alanine and aspartate metabolism, l-kynurenine and l-formylkynurenine represent tryptophan metabolism. *n* = 6, data are presented as mean ± SEM. Statistical significance was assessed using FDR correction. ns: not significant (*p* < 0.05), **p < 0.01, ***p < 0.001).

Interestingly, sarcosine, guanidinoacetate and creatine (Fig. 4) were significantly decreased in S-S-M, but not in P-S-M. Sarcosine is produced from glycine by the enzyme glycine *N*-methyltransferase [KEGG 2.1.1.20] or from creatine by the enzyme creatinase [KEGG 3.5.3.3]. Guanidinoacetate is also produced from glycine by the enzyme glycine amidinotransferase [KEGG 2.1.4.1], and then converted to creatine by the enzyme guanidinoacetate *N*-methyltransferase [KEGG 2.1.1.2].

A significant decrease in these three metabolites could be attributed to the implication of glycine in one-carbon metabolism by donating one carbon to the folate cycle producing more purines and coordinating with glutathione and NADPH biosynthesis, thus maintaining redox balance.^51^ The crucial role of glycine and serine in maintaining redox balance was reported in cancer metabolism as well where the uptake of glycine and serine increased.^51,55^ However, insignificant changes in sarcosine and guanidinoacetate levels and a significant increase in creatine level were shown in P-S-M implying a differential impact of the secretomes of *S. aureus* or *P. aeruginosa* on macrophages. The difference in the metabolic responses of macrophages to the secretomes of *S. aureus* or *P. aeruginosa* can be applied in identifying more selective therapeutic targets in a certain pathway for each bacterial infection.

Another pathway that was altered significantly and differentially upon the bacterial secretome is alanine, aspartate, and glutamate metabolism (Fig. 2A and B). *O*-Acetyl carnitine was significantly decreased in S-S-M but was increased in P-S-M (Fig. 4). Conversely, asparagine was significantly increased in S-S-M but decreased in P-S-M (Fig. 4). Asparagine is a major nitrogen source for the pathogens that infect the skin and human throat. It induces pathogen proliferation and expression of genes including those which are related to metabolism, virulence and growth.^56^ The different asparagine levels observed in response to the two secretomes may indicate distinct macrophage responses to each species and the different strategies that these bacteria use to survive.

Tryptophan metabolism showed a significant perturbation through a significant increase in tryptophan in both S-S-M and P-S-M. Interestingly, l-formylkynurenine showed a significant increase in S-S-M but not in P-S-M, while l-kynurenine showed a significant decrease in P-S-M but not in S-S-M (Fig. 4). Tryptophan is an essential amino acid required for metabolic functions and protein synthesis. It cannot be produced in the body; therefore, it must be supplied in the form of proteins. In the last decades, more focus was directed on the role of tryptophan as an intermediate in the immune system, but its role still not fully understood. However, tryptophan metabolism and inflammatory responses are thought to be associated with several diseases and pathological conditions.^57^ Tryptophan is metabolised through l-kynurenine pathway by the enzyme indoleamine 2,3-dioxygenase (IDO) that is present in macrophages and dendritic cells. IDO is induced under the immune activation condition. IDO breaks l-tryptophan to produce l-formylkynurenine which is consequently metabolised by the enzyme kynurenine formamidase to produce l-kynurenine which is considered as an inflammatory marker.^57^

The increase in the level of l-formylkynurenine in S-S-M could be attributed to the stimulation and activation of macrophages. Unexpectedly, though, l-kynurenine showed no increase in response to the *S. aureus* secretome and a significant decrease in response to the *P. aeruginosa* secretome, suggesting that such effect is due to tolerance and local immunosuppression associated with an enhanced IDO activity which may indicate a stronger impact on this pathway.^58^

Overall, the observed metabolic changes are consistent with known immunometabolic mechanisms. Alterations in arginine and proline metabolism may reflect reduced nitric oxide synthesis, a central antimicrobial effector function of macrophages. Perturbations in tryptophan metabolism, including changes in l-formylkynurenine and l-kynurenine, are indicative of IDO activity, which is known to modulate immune tolerance and inflammatory responses. Similarly, depletion of glycine and related metabolites, together with changes in serine levels, could impair glutathione biosynthesis and thereby compromise redox balance during macrophage activation. These links suggest that the secretome-induced metabolic shifts may have direct consequences for macrophage immune function. However, since our study did not directly measure functional outputs such as cytokine release or phagocytic activity, future work will combine metabolomics with functional immunological assays to define the specific roles of these altered pathways.

**Fig. 5:**
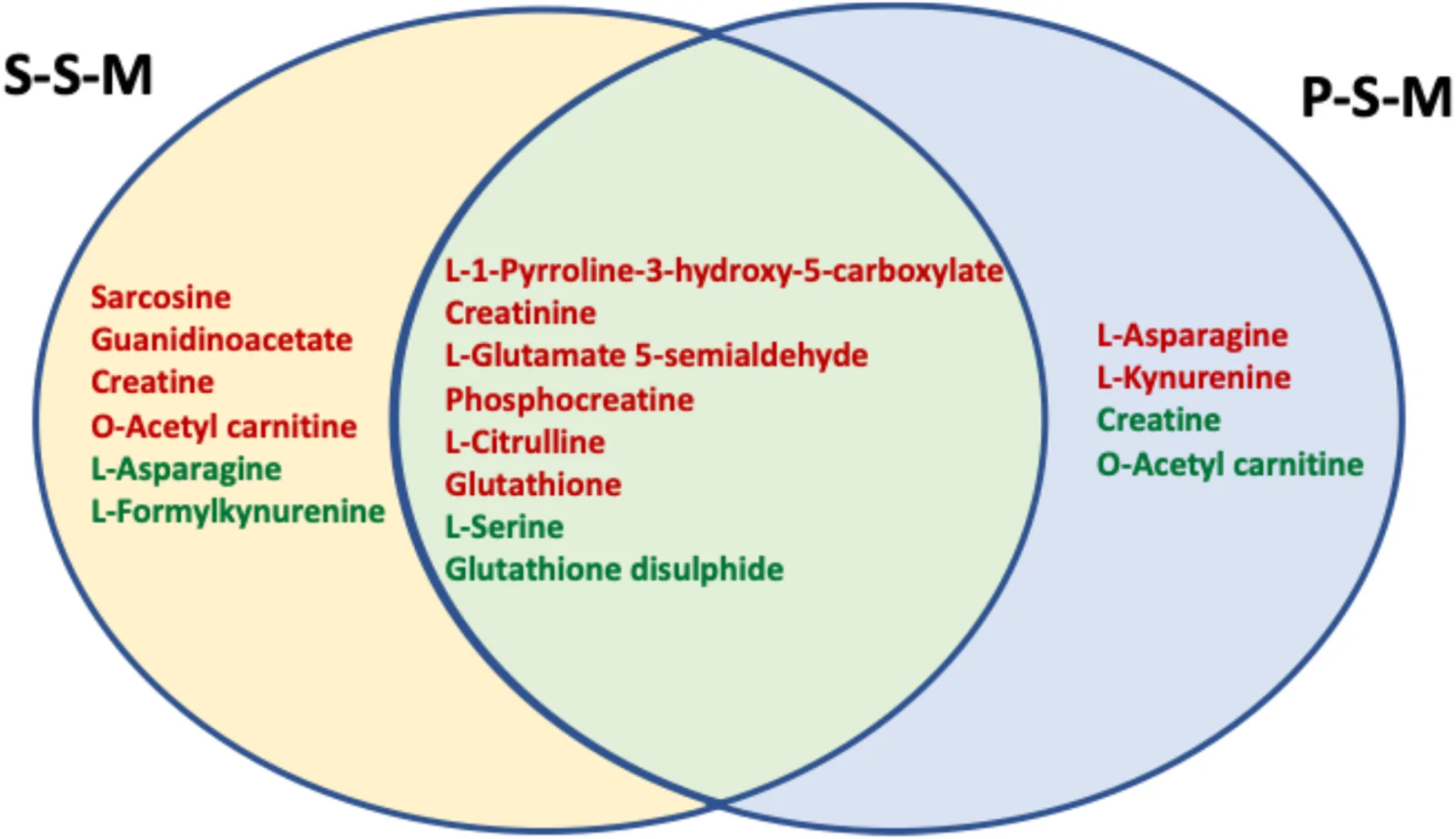
Venn diagram shows significantly altered metabolites in response to S-S-M and P-S-M treatments. Metabolites with similar changes in both conditions are shown in the light green overlap. Metabolites with significantly increased levels are indicated in dark green, while those with significantly decreased levels are shown in red.

Fig. 5 summarises the key similarities and differences in metabolite changes induced by S-S-M and P-S-M treatment. Both bacterial secretomes significantly affected metabolites involved in arginine and proline metabolism (*e.g.*l-1-pyrroline-3-hydroxy-5-carboxylate, creatinine, l-glutamate 5-semialdehyde, l-citrulline, and phosphocreatine), suggesting common mechanisms of immune modulation. However, distinct secretome-specific alterations were also observed. S-S-M treatment led to increased levels of l-asparagine and l-formylkynurenine, along with decreased levels of glycine-related metabolites, including sarcosine and guanidinoacetate. Additionally, *o*-acetylcarnitine and tryptophan pathway intermediates were differentially regulated. In contrast, P-S-M treatment maintained creatine levels, reduced l-asparagine, and modestly perturbed tryptophan metabolism, evidenced by a decrease in l-kynurenine. These different metabolic responses suggest that *S. aureus* and *P. aeruginosa* employ distinct strategies for influencing host immune function and metabolic reprogramming. Specifically, *S. aureus* may enhance redox balance and immune evasion through glycine depletion and active tryptophan catabolism, whereas *P. aeruginosa* may modulate energy use and immune response *via* asparagine regulation. These findings highlight potential pathogen-specific therapeutic targets, such as modulating glycine metabolism in *S. aureus* infections or targeting asparagine pathways in *P. aeruginosa* infections.

**Fig. 6:**
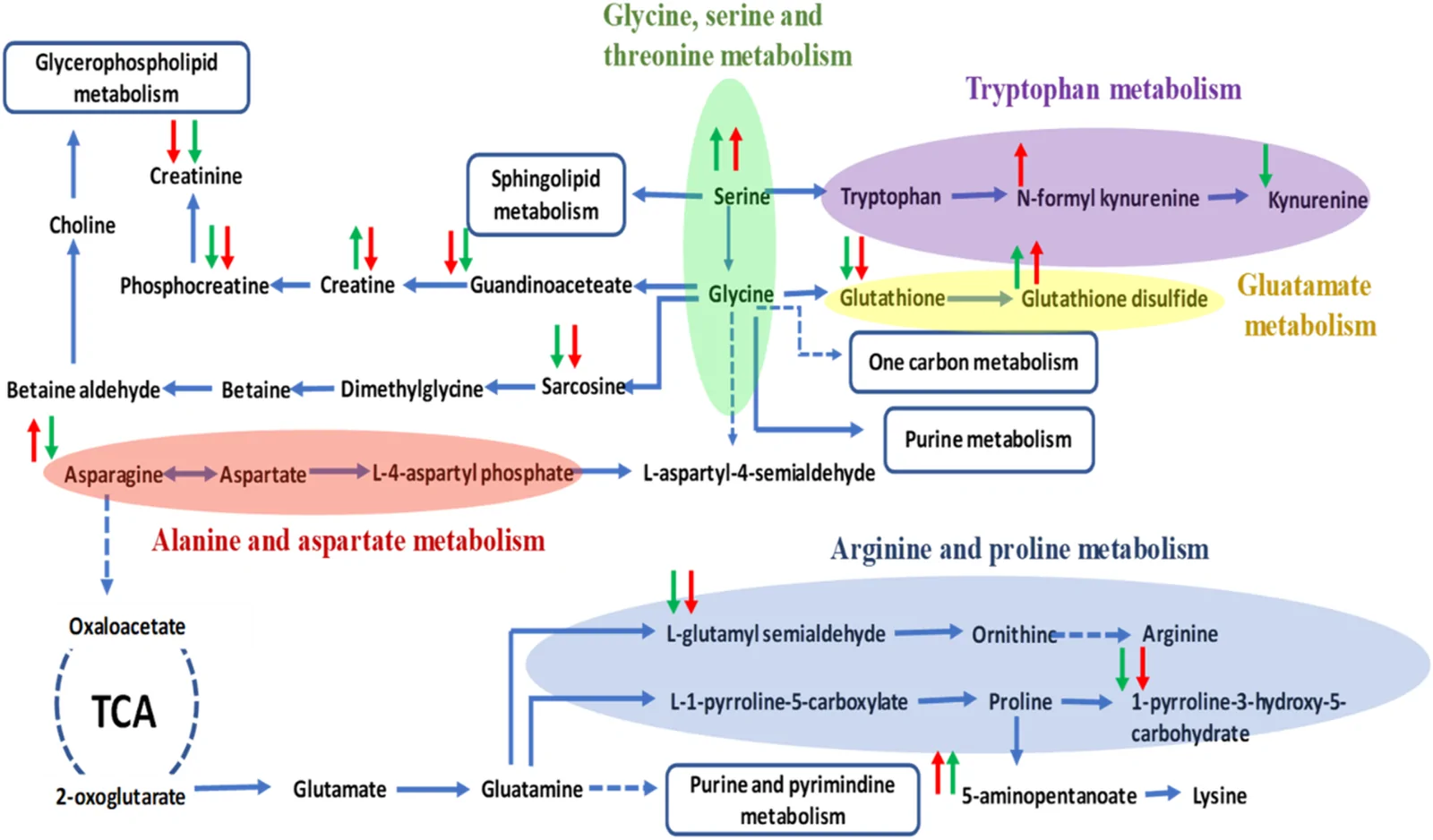
Schematic pathway analysis shows the significantly altered metabolites in their relevant pathways. Upward arrows indicate increased metabolite levels; downward arrows indicate decreases. Red arrows represent changes induced by S-S-M, and green arrows represent those induced by P-S-M.

Fig. 6 represents a schematic pathway analysis of most amino acids related metabolites which were significantly changed in response to the treatment of the secretome from *P. aeruginosa* and *S. aureus* in macrophages.

However, in this study, only one strain of Gram-positive and Gram-negative bacteria were used, and the secretome represents a complex mixture of molecules. Therefore, attribution of the observed effects to specific bacterial factors is limited. Future studies will aim to fractionate bacterial secretomes to identify specific components responsible for the observed effects, and to include multiple clinical strains from both Gram-positive and Gram-negative bacteria. These approaches will improve mechanistic understanding and help determine the extent to which the findings are generalisable across different bacterial species.

Additionally, while THP-1 cells are widely used as a macrophage model in infection and immunometabolism studies,^59^ it is expected that PMA priming *via* protein kinase C activation can influence cellular metabolism and may not fully reflect the behaviour of primary human macrophages. Nevertheless, THP-1 cells offer several advantages that make them appropriate for an exploratory, untargeted LC-MS metabolomics study: they are reproducible, easy to culture, and provide the large cell numbers required for robust metabolite profiling. By contrast, monocyte derived macrophages exhibit high donor-to-donor variability, are difficult to obtain in sufficient quantity, and often yield lower metabolite coverage, which poses challenges at the discovery stage. For these reasons, THP-1 cells were selected as a practical and reproducible model to generate baseline insights into host metabolic responses. We consider this work a discovery-phase investigation intended to identify candidate pathways, with future studies planned to validate these findings in primary macrophages and, ultimately, *in vivo* models.

Beyond such model-related constraints, it is also important to recognise limitations that are inherent to the metabolomics methodology itself. LC-MS metabolomics, like any other analytical approach, has several limitations that stem from technical challenges. These include instrumental limitations, data complexity, accurate identification and fragmentation patterns. Although LC-MS is a powerful tool in metabolomics, its sensitivity can vary significantly. This variability can be overcome through the use of validated methods and regular instrument calibration to ensure accurate quantification across a wide dynamic range of concentrations.^60^ Variations in LC-MS instrumentation across laboratories can also affect reproducibility. Additionally, the vast and complex datasets generated in metabolomics require sophisticated statistical analysis for meaningful interpretation.^61^ However, this challenge is increasingly being addressed by advances in high-quality data pre-processing software.^62^ To address the challenges associated with accurate metabolite identification, future studies should incorporate LC-MS/MS analysis to obtain definitive fragmentation patterns. However, another technical hurdle involves unpredictable fragmentation patterns, which complicate metabolite identification. Although advanced deconvolution methods are being developed to address this issue, there remains a critical need for comprehensive reference standards to expand spectral libraries. Addressing these challenges will further enhance the robustness and reliability of LC-MS metabolomics studies.^60^

## Conclusion

This study demonstrates that LC-MS metabolite profiling is a powerful and effective tool for investigating the metabolic effect of Gram-positive and Gram-negative bacteria on macrophages. The secretomes of *S. aureus* and *P. aeruginosa* exerted distinct metabolic impacts on THP-1 macrophages, particularly in pathways such as glycine and serine metabolism, sphingolipid metabolism, glycolipid metabolism, alanine, aspartate and glutamate metabolism as well as tryptophan metabolism. Despite these differences, both bacterial secretomes induced similar alterations in arginine and proline metabolism, suggesting potential shared mechanisms in modulating the host immune system. Furthermore, the secretome of wild-type *P. aeruginosa* and its mutant variant exhibited comparable impacts on macrophage metabolism, with only a few metabolite variations (*e.g.*, picolinic acid, *N*-acetyl-l-glutamate 5-semialdehyde, 5-guanidino-2-oxopentanoate, and 5-aminopentanoate).

The identification of key metabolites as potential biomarkers for Gram-positive or Gram-negative bacteria offers promising avenues for the development of novel diagnostic strategies for bacterial infections. These biomarkers not only improve the accuracy and early detection of pathogen-specific infections but may also provide insights into specific virulence factors or bacterial strains. Furthermore, understanding the metabolic pathways disrupted by bacterial secretomes enhances our knowledge of the mechanisms underlying bacterial pathogenesis and resistance. This foundational understanding enables the design of targeted drugs capable of overcoming bacterial virulence and resistance. For example, selective targeting of enzymes involved in identified metabolic pathways critical for the immune response or bacterial survival offers a path toward innovative therapeutic approaches. A novel drug could be designed to inhibit or activate these enzymes to disrupt bacterial infections effectively. Supporting this concept, the inhibition of arginase has already shown success in combating *P. aeruginosa* and *S. aureus*.^46,47^

In summary, our results demonstrate that LC-MS metabolite profiling can reveal distinct and overlapping metabolic effects of Gram-positive and Gram-negative bacterial secretomes on macrophages. While these findings do not directly address bacterial pathogenesis or resistance mechanisms, they provide valuable baseline insights into host–pathogen metabolic interactions. Such exploratory data can inform future mechanistic studies aimed at linking metabolic alterations to bacterial virulence and resistance and may ultimately support the identification of novel therapeutic targets.

## Conflicts of interest

The authors declare no competing financial interest.

## Supplementary Material

RA-015-D4RA07202B-s001

RA-015-D4RA07202B-s002

## Data Availability

The original data presented in the study are openly available in the Nottingham Research Data Management Repository at https://rdmc.nottingham.ac.uk/. Supplementary information is available. See DOI: https://doi.org/10.1039/d4ra07202b.
