# Supplementary material for: Metabolomic profiling of host–pathogen interactions: differential effects of Gram-positive and Gram-negative bacterial secretomes on THP-1 macrophage metabolism
Source: RSC Adv. 2025 Oct 24;15(48):40607–18. doi: 10.1039/d4ra07202b (PMC12551783; doi:10.1039/d4ra07202b)
Supplement: RA-015-D4RA07202B-s001 [file RA-015-D4RA07202B-s001.pdf]

Supplementary Information (SI) for RSC Advances.
This journal is © The Royal Society of Chemistry 2025

# Metabolomic profiling of host-pathogen interactions: Differential effects of Gram-positive and Gram-negative bacterial secretomes on THP-1 macrophage metabolism

Alaa Abuawad[a], Manuel Romero[bΨ], Sandra Martinez Jarquin[c], Amir M. Ghaemmaghami[d], Dong-Hyun Kim*[c,e]

[a]Department of Pharmaceutical Sciences and Pharmaceutics, Faculty of Pharmacy, Applied Science Private University, Amman, Jordan

[b]National Biofilms Innovation Centre, Biodiscovery Institute and School of Life Sciences, University of Nottingham, Nottingham, United Kingdom.

[c]Centre for Analytical Bioscience, Advanced Materials and Healthcare Technologies Division, School of Pharmacy, University of Nottingham, Nottingham, NG7 2RD UK

[d]Immunology & Immuno-bioengineering Group, School of Life Sciences, Faculty of Medicine and Health Sciences, University of Nottingham, Nottingham, NG7 2RD UK

[e]College of Pharmacy, Kyungpook National University, Daegu 41566, Republic of Korea

[Ψ]Present Address: Department of Microbiology, Faculty of Biology-Aquatic One Health Research 27 Center (iARCUS), Universidade de Santiago de Compostela, Santiago de Compostela, 15782, 28 Spain

*Corresponding author:

Dong-Hyun Kim
Email: dong-hyun.Kim@nottingham.ac.uk
Tel: +44 (0) 115 74 84697

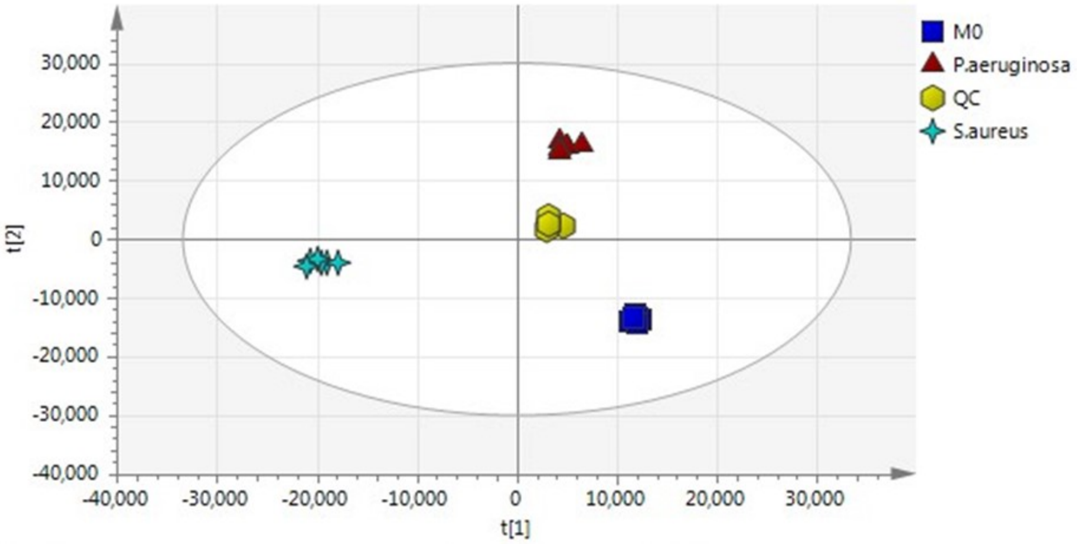


Supplementary Figure 1. PCA scores plot of macrophage extracts after the treatment with the secretome of *S. aureus* (light blue stars) and *P. aeruginosa* (red triangles), their untreated control M0 (dark blue squares) and QC (yellow hexagons).

**A**

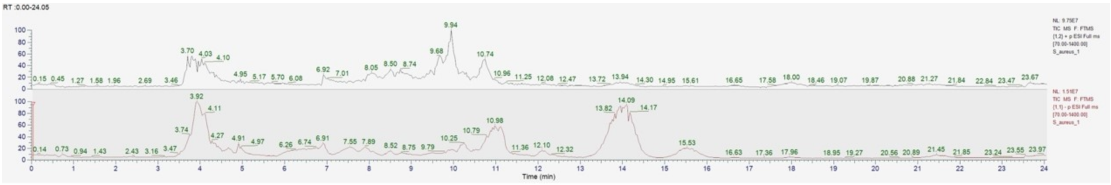

**B**

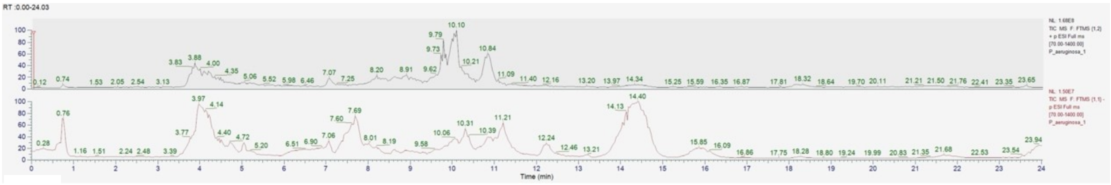

**C**

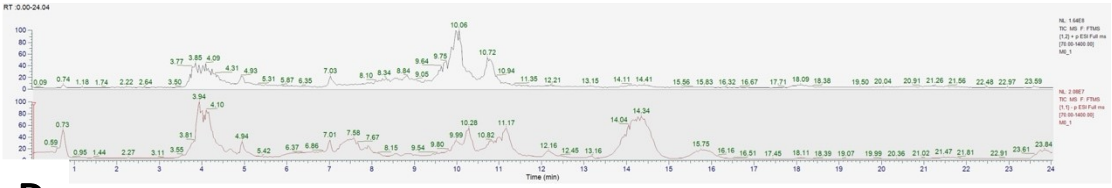

**D**

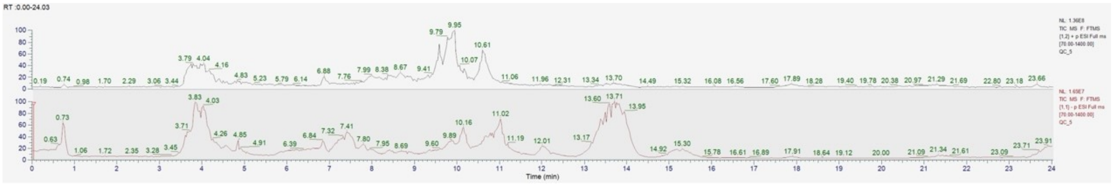

Supplementary Figure 2. Representative total ion chromatograms (TICs) of macrophage extracts after the treatment with the secretome of *S. aureus* (A) and *P. aeruginosa* (B), compared with their untreated controls (M0; C) and QC (D). In each panel, the top TIC corresponds to positive ionisation mode, and the bottom TIC represents negative ionisation mode.

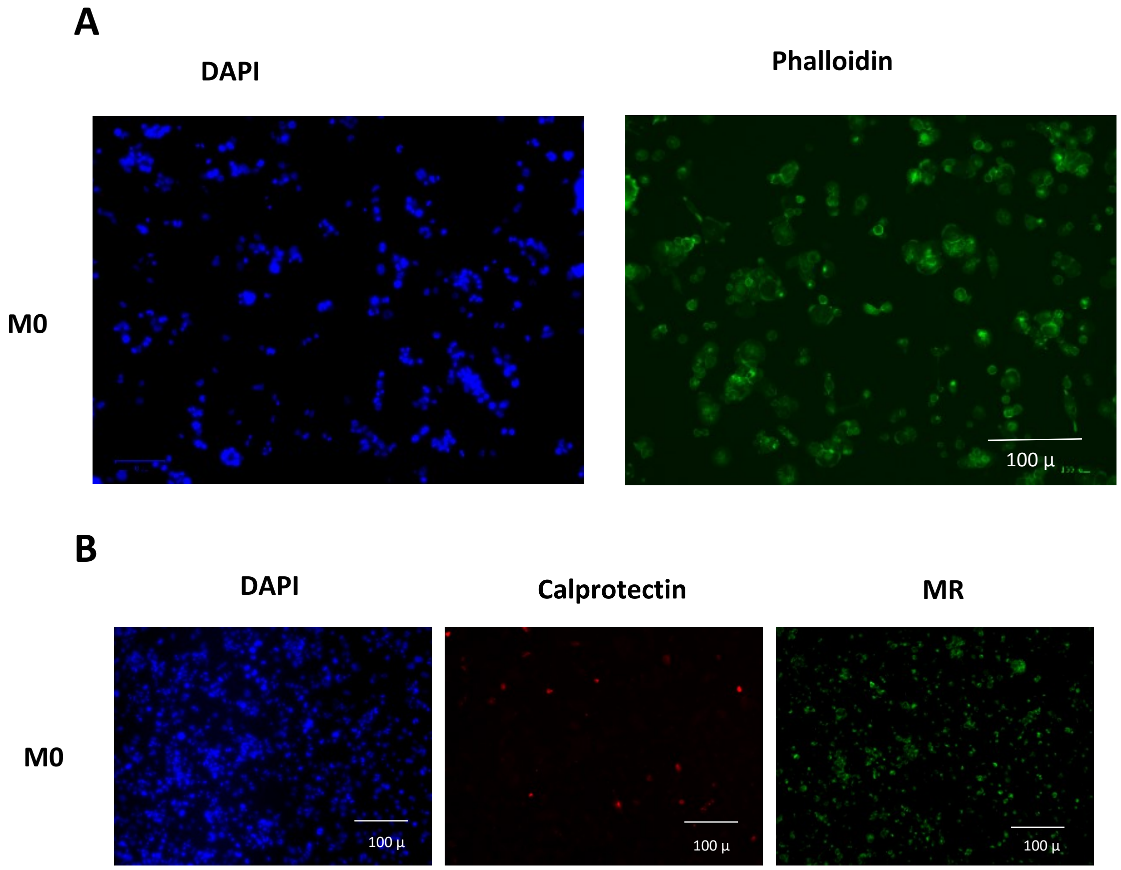


Supplementary Figure 3. (A) Morphology of differentiated THP-1 cells visualised by staining with phalloidin (green) for F-actin and DAPI (blue) for nuclei. Images were acquired at 10× magnification; scale bar = 100 µm. (B) Immunofluorescence staining showing expression of surface markers in differentiated THP-1 cells: mannose receptor (MR; green), calprotectin (red), and nuclei (DAPI; blue). Scale bar = 100 µm.

**A**

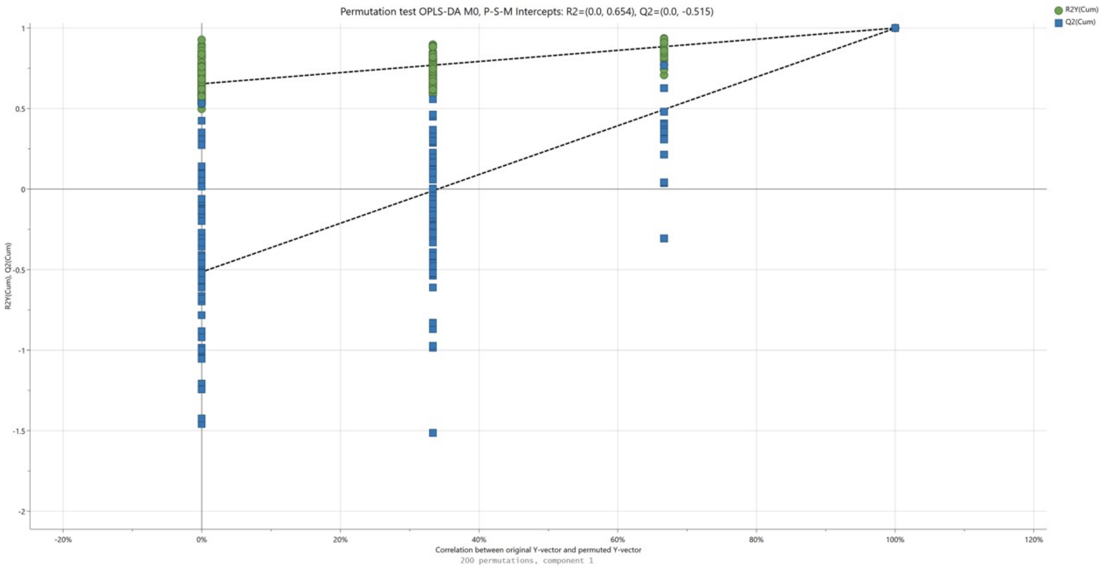


**B**

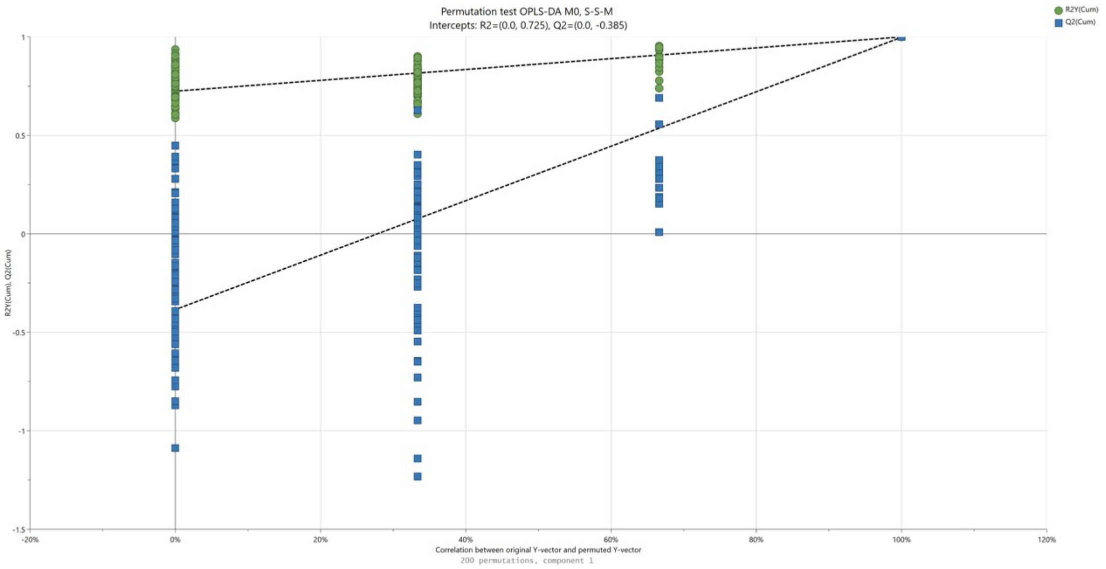


Supplementary Figure 4. Permutation tests with 200 random permutations were performed on each OPLSDA model: (A) P-S-M and (B) S-S-M, each compared with M0. The permutation plots indicate that both models are valid, as $Q^2$ values (blue squares) from the permuted tests (bottom left) are lower than the corresponding original values (top right).
